# Vertical transfer and functional characterization of cotton seed core microbiome

**DOI:** 10.3389/fmicb.2023.1323342

**Published:** 2024-01-09

**Authors:** Chongdie Wu, Xin Zhang, Yongbin Fan, Jingyi Ye, Lingjun Dong, YuXiang Wang, YinZheng Ren, HongHong Yong, Ruina Liu, Aiying Wang

**Affiliations:** ^1^College of Life Sciences, Shihezi University, Shihezi, China; ^2^Xinjiang Production and Construction Corps, Key Laboratory of Oasis Town and Mountain-basin System Ecology, Shihezi, China

**Keywords:** high-throughput sequencing, cotton microbiome, vertical transfer, seed endophytic microbiome, functional traits

## Abstract

**Introduction:**

Microbiome within plant tissues is pivotal for co-evolution with host plants. This microbiome can colonize the plant, with potential transmission via seeds between parents and offspring, affecting seedling growth and host plant adaptability to the environment.

**Methods:**

We employed 16S rRNA gene amplicon analysis to investigate the vertical distribution of core microbiome in cotton seeds across ecological niches [rhizosphere, root, stem, leaf, seed and seed-P (parental seed)] of the three cotton genotypes.

**Results:**

The findings demonstrated a significant decrease in microbiome diversity and network complexity from roots, stems, and leaves to seeds. The microenvironment exerted a more substantial influence on the microbiome structure of cotton than the genotypes. The core endophytic microorganisms in cotton seeds comprised 29 amplicon sequence variants (ASVs) affiliated with *Acidimicrobiia*, *Alphaproteobacteria*, *Bacilli*, *Bacteroidia*, *Clostridia*, *Gammaproteobacteria*, *and* unclassified_*Proteobacteria*. These vertically transmitted taxa are widely distributed in cotton plants. Through 16S rRNA gene-based function prediction analysis of the cotton microbiome, we preliminarily understood that there are potential differences in metabolic capabilities and phenotypic traits among microbiomes in different microhabitats.

**Discussion:**

In conclusion, this study demonstrated the crucial role of the microenvironment in influencing the cotton microbiome and offered insights into the structures and functions of the cotton seed microbiome, facilitating future crop yield enhancement through core seed microbiome regulation.

## Introduction

The plant microbiome plays a pivotal role in plant health and forms a large biological system known as the holobiome (Vandenkoornhuyse et al., 2015), interacting with plants at various physiological, ecological, and evolutionary levels. This interaction commences at the seed stage, where microbiomes, particularly seed-endophytic microorganisms, significantly influence seedling growth vigor and the environmental adaptation of the plant (Frank et al., 2017). Seed-endophytic bacteria engage in intricate ecological relationships with plant hosts, including symbiosis, co-evolution, and benign parasitism. Importantly, plants selectively transmit beneficial taxa from one generation to the next (Ferreira et al., 2008; Hardoim et al., 2011; Truyens et al., 2014; Sánchez-López et al., 2018).

Situated in a warm temperate and inland region, Xinjiang is renowned for its ample heat and sunlight, conditions that are particularly conducive to cotton cultivation. Cotton (*Gossypium* spp.) holds a pivotal role in global agriculture, not merely as a strategic material integral to national economies but also as a significant contributor to economic benefits. Its wide-ranging applications span textiles, oil production, and the medical industry (Predeepa et al., 2022; Zhang et al., 2023). Leveraging the unique geographic advantages of Xinjiang, conducive to optimal cotton growth, this region accounts for over 80% of China’s cotton yield, thereby constituting nearly a quarter of global production (Ju et al., 2021). Cotton is vital for regional economic growth and the preservation of ecological balance (Adham et al., 2020). Nonetheless, the sustainability of cotton production is increasingly challenged by climate change, pest infestations, and various environmental stressors (Nelson, 2004). Consequently, rigorous research into the cotton seed microbiome is imperative for augmenting the crop’s resilience against such adversities.

The current study focuses on fundamental research into the cotton microbiome, particularly concerning rhizosphere and endophytic fungi (Nelson, 2018; Da Silveira et al., 2019; Hone, 2021; Bai, 2022; Hou, 2023), yet research on the cotton endophytic bacterial microbiome remains limited. Studies of plant microbiome have shown that research on rice microbiome reveals the origins of seed endophytes, the composition of core microbiome, and the interaction patterns between microorganisms and host plants (Walitang et al., 2019; Zhao, 2019; Zhang et al., 2022), providing an important reference framework for our study on the formation and function of cotton seed microbiome. Understanding the composition and mechanisms of the seed microbiome allows us to better infer and analyze potential characteristics of the cotton seed microbiome, particularly in response to various stresses. Recognizing the importance of priority effects, optimizing the seed microbiome methods holds significant potential for plant breeding and crop improvement (Mitter et al., 2017; Chesneau et al., 2020). Identifying the core plant microbiome serves as a valuable resource in enhancing defenses against environmental stresses and plant diseases (Gopal et al., 2013). Therefore, this study aims to fill the research gap in the cotton seed microbiome, which is crucial for improving cotton’s adaptability and productivity.

This study aimed to achieve the following specific objectives: (1) investigate the structure and functionality of microbiome associated with cotton, (2) identify the core taxa within cotton seed microbiome, and (3) examine the vertical diffusion of seed microorganisms and their distribution across various plant parts throughout the growth stages of cotton.

## Materials and methods

### Experimental setup and sampling

**FIGURE 1:**
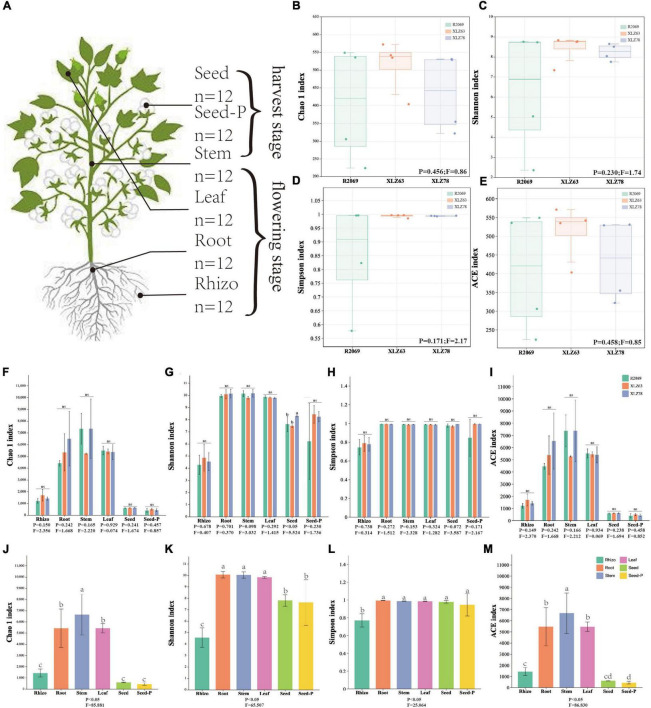
Alpha diversity of cotton-associated microbiomes. **(A)** Samples were collected from five microhabitats. Rhizo, rhizosphere; Root, root endosphere; Stem, stem endosphere; Leaf, leaf endosphere; Seed, offspring seed endosphere; Seed-P, parental seed endosphere. n represents the number of samples. **(B–E)** Chao 1 index, Shannon index, Simpson index, and ACE index of the microbiota in the parental seed endosphere of three genotypes. R2069, Zhong R2069; XLZ63, Xinluzao 63; XLZ78, Xinluzao 78. Horizontal bars within boxes represent medians. Tops and bottoms of boxes represent the 75th and 25th percentiles, respectively. Statistical significance was determined using ANOVA and *T*-test. Lowercase letters denote statistically significant differences at the 95% confidence interval (*p* < 0.05). **(F–I)** Chao 1 index, Shannon index, Simpson index, and ACE index of the microbiota in different microhabitats from three cotton genotypes. ns indicates no statistically significant difference based on the T-test (*p* < 0.05). **(J,M)** Chaol index, Shannon index, Simpson index, and ACE index of the microbiota in different microhabitats. Means with the same letters are not statistically different based on the *T*-test (*p* < 0.05). Indices (*F* value and *p*-value) are displayed at the bottom of each graph.

In Shihezi City, Xinjiang, located at 86°3’52.884” E and 44°18’36” N, we cultivated three cotton genotypes: R2069, XLZ63, and XLZ78. Complete cotton plants and corresponding rhizosphere samples were collected at the flowering stage, followed by seed collection at harvest. Each of the three distinct genotypes was planted in quadruplicate, with each plot measuring 18 m^2^ (3 m × 6 m) and managed with a fertilization regimen of 40 kg of nitrogen, 25 kg of phosphate (P_2_O_5_), and 20 kg of potash (K_2_O) per mu. Of the nitrogen fertilizer, 25% was used as base fertilizer, with the remaining 75% applied in stages. For phosphate and potash fertilizers, 70% and 50%, respectively, were used as base fertilizers, with the remainder used as top dressing. Approximately eight rounds of top dressing were conducted throughout the growing period, following the ‘one water, one fertilizer’ management strategy. To comprehensively assess the microbiomes associated with these cotton genotypes, we extracted total DNA from a broad range of samples, comprising 12 rhizosphere samples, 12 root samples, 12 stem samples, 12 leaf samples, 12 seed samples, and 12 seed-P (parental seed) samples, as illustrated in Figure 1A. Each sequenced tissue sample consisted of a composite from four individual plants within the same plot. Using sterile scissors, we cut the roots of the cotton plants and then centrifuged the cuttings at 12,000 rpm for 10 min to collect the rhizosphere, defined as the soil layer within 1–2 mm of the root. Each seed sample was standardized to 10 g. Root, stem, leaf, and seed samples were initially rinsed with tap water to remove the majority of soil and debris. Subsequently, these samples were immersed in 75% ethanol for 1 min for disinfection, followed by a 10-min soak in a 50 g/L sodium hypochlorite solution. Afterward, the samples were thoroughly rinsed three times with sterile water to remove all residual disinfectants. Finally, the surface moisture was gently blotted with sterile paper towels, and the samples were placed in a sterile environment to dry. Subsequently, these samples were individually stored at −80°C for further processing and subsequent analysis.

The measurements of organic matter (OM), alkali-hydrolyzable nitrogen (AN), available potassium (AK), available phosphorus (AP), electrical conductivity (EC), and pH in cotton rhizosphere soil were all conducted in accordance with the soil quality guidelines (Hill and Sparling, 2009).

### Illumina and 16S rRNA gene sequencing

Total genomic DNA was extracted using a DNeasy PowerSoil Kit (MP Biomedicals, Germany). The extracted DNA was characterized using 1.0% agarose gel electrophoresis and quantified using a NanoDrop One spectrophotometer (Thermo Scientific, USA). The DNA samples were stored at −80°C for future investigations.

In terms of gene library construction, we used the universal primers 335F and 769R to amplify the V3–V4 regions of the 16S rRNA gene, which predominantly amplifies bacterial DNA in plant samples, thereby significantly reducing the likelihood of amplifying plant, archaeal, and other eukaryotic DNA (Dorn-In et al., 2015). Polymerase chain reaction (PCR) was performed using the Phanta Max Master Mix Kit P515 (Vazyme, China) under predefined thermocycling conditions. The procedure commenced with an initial denaturation step at 95°C for 3 min. The amplification comprised seven cycles, each including a denaturation step at 95°C for 45 s, followed by annealing at 65°C for 1 min with a gradual cooling rate of 2°C per cycle, and an extension step at 72°C for 90 s. This was followed by 30 consecutive cycles of stable-state amplification, comprising a 15-s denaturation step at 95°C, a 30-s annealing step at 50°C, and a 30-s extension step at 72°C. The amplification was completed with a final 5-min extension step at 72°C. The amplification products were validated using 1.0% agarose gel electrophoresis. High-throughput sequencing was conducted by BMKCloud Co. using Illumina HiSeq P250 technology, which yielded 250 bp paired-end reads.

In this study, raw data were initially subjected to demultiplexing and quality filtering using Trimmomatic (Bolger et al., 2014) (version 0.33), with quality filtering employing a 50 bp sliding window to trim the trailing bases when the average quality within the window fell below 20. Subsequently, primer sequences were identified and removed with Cutadapt (Martin, 2011) (version 1.9.1) using a maximum mismatch rate of 20% and a minimum overlap of 80%. Paired-end reads were then merged using USEARCH (Edgar, 2016) (version 10) and chimeric sequences were identified and removed with UCHIME (Edgar et al., 2011) (version 8.1). Afterward, the quality-controlled data were denoised using the DADA2 (Callahan et al., 2016; Bolyen et al., 2019) method within QIIME2 (Bolyen et al., 2019) (version 2020.6), setting the threshold to 0.005% of all sequencing reads to filter out ASVs. From a sample set that included 12 rhizospheres, 12 roots, 12 stems, 12 leaves, 12 seeds, and 12 seeds-P, 12,605,376 raw reads were obtained, with an average of 165,637 high-quality reads per sample. After quality trimming, each sequence was classified using the classify-sklearn tool within QIIME2 based on the Naive Bayes classifier (Wang et al., 2007), referencing the SILVA small subunit rRNA database (version 138) with a confidence threshold set at 0.7 (Quast et al., 2012). After excluding ASVs classified as mitochondrial, chloroplast, or archaeal, 123,828 bacterial ASVs were successfully identified from 8,261,802 non-chimeric reads, encompassing 50 bacterial phyla, 131 classes, and 2645 genus. α-diversity analyses were conducted at the ASV level based on Chao 1, Shannon, Simpson, and ACE diversity indices. In addition, beta diversity analyses were performed at the ASV level using Bray-Curtis dissimilarity. The asymptotic behavior of the sample dilution curves confirmed the representativeness of the sampling depth. All diversity analyses were completed on the BMKCloud microbiome diversity analysis platform (BMKCloud, 2022).

### Species taxonomic composition and distribution of the seed microbiome

Taxonomic composition was assessed at the class and genus levels across six distinct microhabitats: rhizosphere, root, stem, leaf, seed, and seed-P. The top 20 most abundant classes and genera were also identified. The filtering criteria included a minimum count threshold of 1, presence in >10% of the samples, and consolidation of taxa with counts <10. This taxonomic analysis was performed on 50 bacterial phyla and 2645 genera. At the phylum level, taxa with ASV counts below 10 were categorized into the “other”. At the genus level, the first 50 dominant bacteria were identified, with the remainder classified as “other”, whereas only the top 20 positions were explicitly labeled. The microbiome distributions were visually represented using stacked histograms created using the ggplot2 tool in R (Wickham, 2016). To investigate the vertical distribution of microorganisms during the seed-to-seedling transition, a comparative analysis was conducted between microbiomes in different tissues of germinating seeds and seedlings. These microbiomes were subsequently compared to the bacterial and fungal populations present in the seeds before sowing. This study postulated that if there is a transfer of ASVs from seeds to various plant tissues (including roots, stems, leaves, seeds and seeds-P) of both seedlings and mature host plants, there should be a similarity in the microbiome composition across these tissues. Community dissimilarity was quantified using the vegan package in R programming language. The Jaccard dissimilarity index was used to measure the dissimilarity between the two communities by calculating the ratio of shared ASVs to the total number of ASVs, regardless of their individual abundances. To investigate the phenomenon of microbiome migration from seeds to host plants during the planting process, we initially identified ASVs present in both seeds and various cotton plant tissues (leaves, stems, roots, and progeny seeds). These specific ASVs were denoted as “seed source ASVs”. The relative proportion of seed-source ASVs was estimated by determining the ratio of the number of seed-source ASVs to the total number of ASVs in each tissue. Bar graphs were used to visually depict the composition of seed-derived ASVs, presenting their cumulative relative abundance.

### Selection of core endophytes in cotton seeds

Common ASVs across all plant microhabitats were visualized using Venn diagrams with the InteractiVenn1 (Heberle et al., 2015). The term “common ASVs” refers to ASVs that are identified in all samples within each group chamber. The core ASVs present in cotton were determined using frequency analysis with a 70% threshold. The SourceTracker package (Knights et al., 2011) (version 1.0.1) in the R software (version 3.1.1) was used to analyze and identify the microbiome sources of cotton progeny seeds. This analysis employed a Bayesian algorithm that explores microbiome origins (source) in the target sample (sink), using the seed sample as the sink and the seed-P, along with each microhabitat samples, as the sources. Predictions were based on the distribution of community structures in the source and sink samples, enabling the estimation of the proportion of progeny seed composition originating from distinct cotton tissue parts and progeny seeds.

### Network construction

We conducted a comprehensive analysis of the bacterial community networks associated with cotton within six distinct microhabitats. Networks were constructed at the ASV level using the SparCC algorithm, and significant interactions between ASVs (*p* < 0.05) were identified in the network from ASVs with relative abundance greater than 0.01%. These significant interactions indicate relationships of interdependence or influence observed within the microbial community. Revealing potential functional interactions within the microbial community is of great importance for understanding the structure and function of these communities. ASVs present in at least 25% of the data were evaluated using the construction criteria determined through random matrix theory (RMT). Network analysis, including correlation analysis and modularity identification, was performed using the Molecular Ecological Network Analysis (MENA) platform (Deng et al., 2012; Feng et al., 2022), accessible at http://ieg2.ou.edu/MENA/, as described by Deng and Feng. Network visualization was executed utilizing Gephi 0.9.2 software (Bastian et al., 2009), while network modularity assessment relied on an optimized greedy method. Furthermore, intramodular connectivity (Zi) and intermodular connectivity (Pi) were calculated for each network node to identify the potential keystone species. Nodes were classified into four categories based on their network structure: module centers, network centers, links, and peripheral nodes. Graphics in this study were generated using ggplot2 (version 3.3.2) (Wickham, 2016).

### Functional gene prediction

To comprehensively elucidate the predominant microbiome biological processes in various cotton tissues, including the roots, stems, and leaves, we employed two distinct functional prediction methods. The first method involves a manual correlation of prokaryotic taxa, such as genera, with metabolic or ecological functions, such as nitrification, denitrification, or fermentation. This correlation relies on information derived from literature concerning cultured representative microorganisms. To facilitate this process, we utilized the FAPROTA database (version 1.2.6) as outlined by Louca et al. (2016). A Python script provided by FAPROTAX was used to convert the ASV table into a putative functional table. This conversion was based on the taxa detected in the samples and their corresponding functional annotations in the FAPROTAX database.

To achieve a more comprehensive prediction of microbiome phenotypes, we used BugBase (Ward et al., 2017). This tool can analyze 16S rRNA gene sequence data to predict a range of phenotypic characteristics, including redox conditions (aerobic or anaerobic), presence or absence of mobile elements, biofilm formation, Gram stain response (positive or negative), potential pathogenicity, and stress resistance.

Functional and phenotypic predictions obtained from the two techniques were further evaluated by comparison using the Wilcoxon rank-sum test within the Statistical Analysis of Metagenomic Profiles (STAMP v2.1.3) (STAMP, 2015) software. The main objective of this integrated approach was to provide a comprehensive perspective on the functional diversity and potential ecological roles of microbiomes within various cotton tissue types.

### Statistical analysis and visualization

All statistical analyses were performed using R software (R Core Team, 2013) (version 2023.06.1 + 524) as the default platform, unless otherwise specified, with a predetermined statistical significance threshold of alpha = 0.05. Adjustments for multiple hypothesis testing were conducted using the false positive detection rate (FDR) when applicable. Sparsity corrections were conducted for the α-diversity analysis. The microbiome software package for R (Lahti and Shetty, 2019) (version 1.9.13) was used to obtain Shannon and Simpson diversity values using its alpha function. For microorganisms with relative abundances exceeding 0.5%, their distributions were visualized utilizing the ggplot2 package for R (Wickham, 2016) (version 3.2.1). Assessment of beta diversity among samples employed the weighted UniFrac algorithm, which was based on ASV abundance and composition. Finally, the Principal Coordinate Analysis (PCoA) tool in R (Gower, 1966) was employed for graphical analysis of sample similarities.

## Results

### Quality metrics of sequencing analysis

To methodically investigate cotton-associated microbiomes, we performed DNA amplification and sequencing targeting the V3–V4 region of the bacterial 16S rRNA gene. From a comprehensive dataset comprising 72 samples, a total of 12,605,376 raw reads were generated. These samples included 12 rhizosphere soil samples, 12 root samples, 12 stem samples, 12 leaf samples, 12 seed samples, and 12 seed-P samples. The average number of non-chimeric reads per sample was 114,747 (Supplementary Table 1). After rigorous quality control to eliminate chimeric and plant-derived reads, 8,261,802 non-chimeric reads were retained. These reads were subsequently classified into 123,828 bacterial ASVs and are detailed in Supplementary Table 2. The analysis of 16S rRNA ASVs included their distribution within a diverse spectrum of bacterial phyla and genera, with 50 phyla and 2,645 genera identified (Supplementary Table 2). To assess the comprehensiveness of the sequencing process, a dilution study was conducted. The results of this analysis confirmed the adequacy of our sequencing methodology for capturing the true microbiome diversity present in the samples (Supplementary Figure 1A).

### Diversity and drivers of the cotton microbiome

**FIGURE 2:**
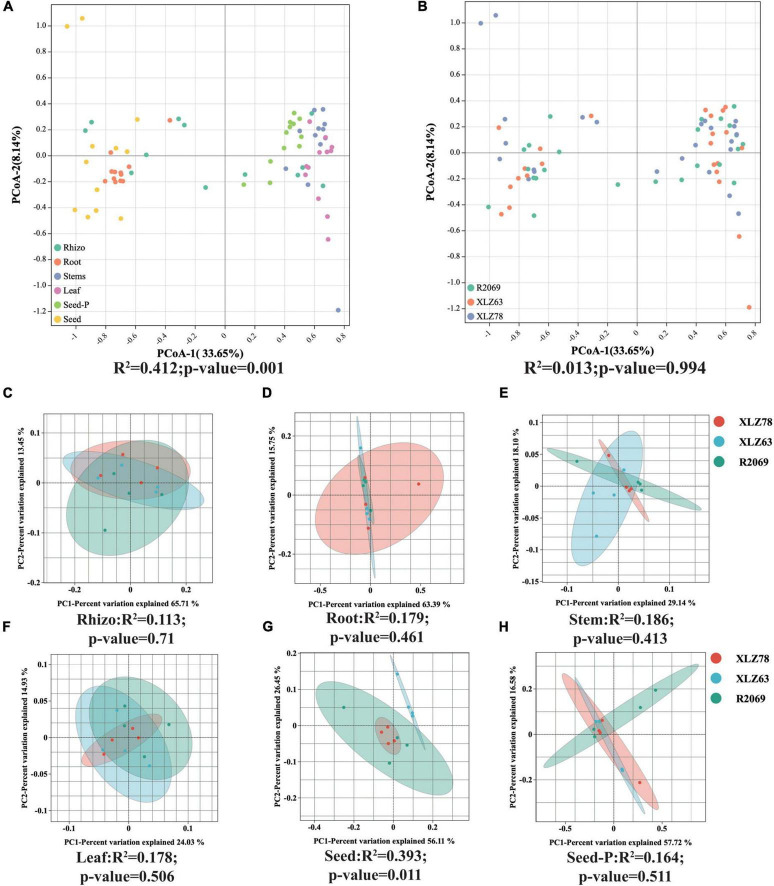
Beta diversity patterns of cotton-associated microbiomes. **(A)** Principal Coordinate Analysis (PCoA) using Bray-Curtis distances, examining differences in microbial communities unconstrained by cotton genotypes. PCo-1 and PCo-2 represent the first and second principal components of the PCoA analy- sis, respectively. **(B)** PCoA of microbial communities in each cotton genotype. Microhabitats and genotypes are depicted by ellipses and point shapes. **(C–H)** show PCoA plots of microbial communities from different genotypes within each microhabitat. Significance of microbial community dissimilarities among different groups is based on PERMANOVA tests. Indices (R2 and *p*-value) are displayed at the bottom of each graph.

We conducted a comprehensive investigation of cotton-associated microbiome diversity using α-diversity indices (abundance and richness, using the Chao1 and ACE indices). The diversity and evenness were assessed using the Shannon and Simpson indices for each sample. Microbiomes from different cotton genotypes were analyzed separately in the rhizospheres, roots, stems, leaves, seeds and seeds-P. The results indicated no significant differences in the alpha diversity of endophytic bacteria within seeds-P across different genotypes (Figures 1B–E and Supplementary Table 3). Additionally, the α-diversity indices of endophytic microbiomes in the rhizospheres, roots, stems, leaves, seeds and seeds-P of various cotton genotypes did not exhibit significant differences overall (Figures 1F–I and Supplementary Figure 2). This suggests that microbiome diversity in different ecological niches of cotton was minimally affected by the genotype. Conversely, microbiome α-diversity within each cotton site (rhizosphere, root, stem, leaf, seed and seed-P) exhibited significant variation across microhabitats (Figures 1G–M and Supplementary Table 3). These results suggest that microhabitats, rather than cotton genotypes, primarily dictate the microbiome diversity. To further confirm this observation, we performed a principal coordinate analysis (PCoA) to elucidate the underlying relationships among the factors (Figure 2). The PCoA results strongly indicated substantial differences in the distribution of the cotton microbiome across ecological niches (e.g., rhizospheres, roots, stems, leaves, seeds and seeds-P) (Figure 2A), with these distinctions not being attributed to cotton genotypes (Figure 2B). Microbiomes from various ecological niches exhibited convergence in rhizospheres, roots, stems, leaves, seeds and seeds-P, although significant overlap was observed in the internal microbiome diversity of roots, stems, and leaves (Figures 2C–H). We evaluated six physicochemical characteristics of rhizosphere soil samples from three cotton genotypes and observed no significant differences among these parameters, indicating that the soil fertility is comparable among the three genotypes (Supplementary Figure 1B).

Microhabitats have emerged as the predominant determinants of diversity within the cotton microbiome, revealing a distinct spatial distribution pattern in microbiome diversity associated with cotton.

### Taxonomic composition and dynamics of the cotton microbiome

**FIGURE 3:**
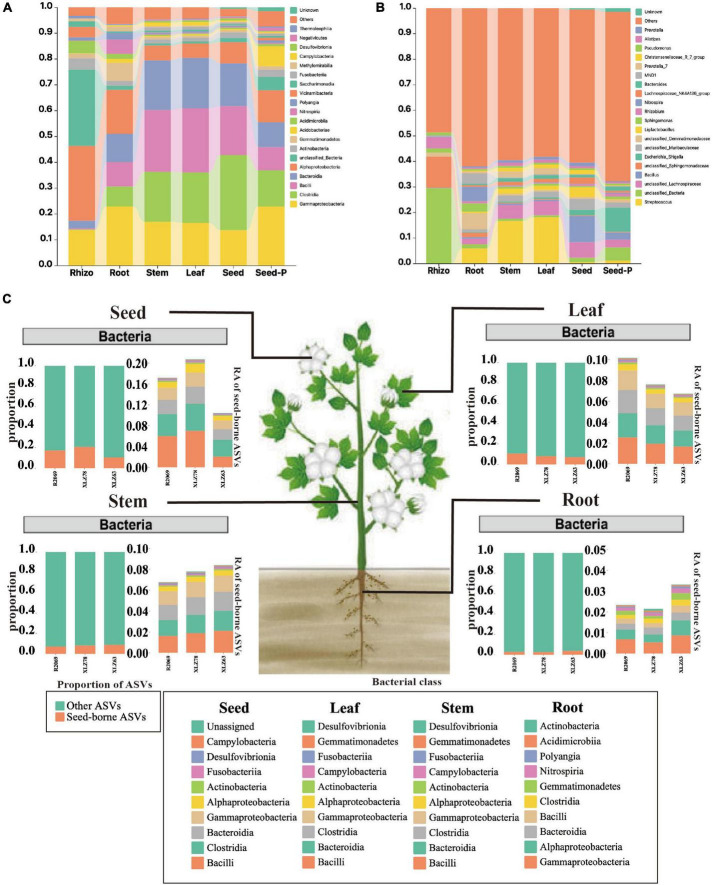
Taxonomic composition of cotton-associated microbiomes in each microhabitat. **(A)** Histograms of class abundances present in the rhizosphere (Rhizo), root endosphere (root), stem endosphere (stem), leaf endosphere (leaf), offspring seed endosphere (seed), and parental seed endosphere (seed-P). **(B)** Relative abund ances of the most prevalent genera in different microhabitats. **(C)** Distribution of seed-borne bacterial communities in compartments of field-grown cotton plants. The distributions of seed-borne bacterial ASVs (amplicon sequence variants) were investigated in different plant compartments: leaf, stem, root, and seed endospheres. In each compartment, the proportion of seed-borne ASVs was calculated by dividing the number of seed-borne ASVs by the total number of ASVs in that compartment. The proportions of seed-borne ASVs are represented by magenta-colored bars, while the remaining portions are indicated by cyan-colored bars. The taxonomic composition of seed-borne ASVs in plant compartments is shown at the class level. Each color on the bar plots represents a bacterial class. The chart shows the top 10 class in terms of relative abundance. The raw data on bacterial relative abundances can be found in Supplementary Table 6. RA indicates relative abundance.

To comprehensively investigate the taxonomic characteristics and dynamics of the cotton microbiome under varying conditions, we conducted a systematic analysis across multiple microhabitats, including rhizospheres, roots, stems, leaves, seeds and seeds-P. Figure 3, Supplementary Figure 3, and Supplementary Table 4 illustrate different degrees of enrichment or depletion within the cotton microbiome at both the phylum and genus taxonomic levels. The analysis revealed *Gammaproteobacteria* as the dominant taxon across most microhabitats, constituting over 13% of the ASVs. Nevertheless, the abundance and distribution of *Gammaproteobacteria* exhibited significant variation among microhabitats (Supplementary Figure 3 and Supplementary Table 4). For example, *Gammaproteobacteria* accounted for 22.9% and 18.4% of ASVs in seeds-P and roots, respectively, whereas *Alphaproteobacteria* were the most abundant in rhizospheres, reaching 29.6% of ASVs. Certain bacterial groups (class), such as *Clostridia*, *Bacilli*, and *Bacteroidia*, showed a gradual increase from seeds-P to roots, stems, leaves, and seeds. Their abundance was relatively low in rhizosphere, but progressively increased within plant tissues, particularly in stems, leaves, and progeny seeds (Figures 3A–C and Supplementary Table 4). Particularly noteworthy is the change in microbiome composition from seed-P to seeds, with a marked decrease in the relative abundance of *Gammaproteobacteria*, and an increase in the relative abundance of *Clostridia*, *Bacilli*, and *Bacteroidida*.

In addition, *Bacilli* were identified as the predominant bacterial group during cotton development from root to seed, accompanied by a gradual decline in unclassified_*Sphingomonadaceae* and certain unclassified_*Bacteria* genera within the *Alphaproteobacteria*. This pattern indicated a significant influence of seed-derived microorganisms on the aboveground portion, whereas rhizosphere microbiomes were predominantly affected by the field environment.

### Key taxa and co-occurrence networks of the bacteria

**FIGURE 4:**
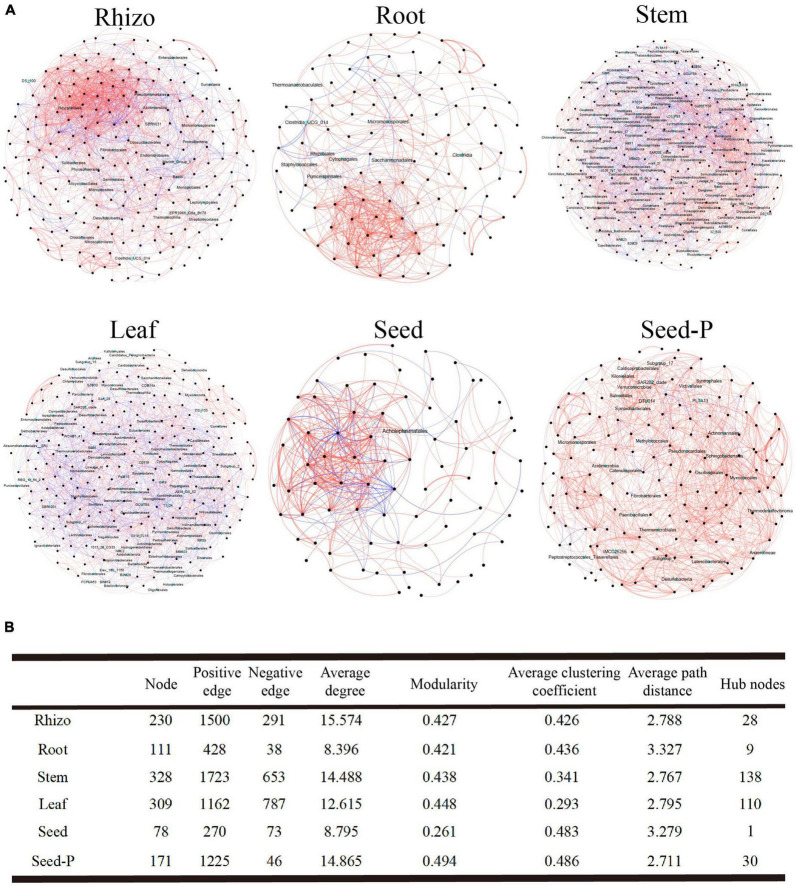
Microbial association network diagram. **(A)** This section describes the co-occurrence and mutual exclusion relationships among cotton-associated microbiomes across the soil-plant continuum. The SparCC algorithm was used to calculate the network at the ASV (Amplicon Sequence Variants) level with an abundance greater than 0.0 I % and p < 0.05. Each node represents a single ASV, and hub nodes are indicated in cyan. The color of the edges represents the type of interaction: red for positive or co-occurrence relationships, and blue for negative or mutual exclusion relationships. **(B)** This part displays the major topological properties of co-occurrence networks for each microhabitat. Detailed data is provided in Supplementary Table 5.

To comprehensively investigate the bacterial interactions within cotton microbiomes across distinct microhabitats, including rhizosphere, roots, stems, leaves, seeds, and seeds-P, we constructed six bacterial co-occurrence networks (Figure 4). Overall, these networks predominantly exhibited positive correlations among the bacterial taxa. A detailed analysis of network topological parameters revealed that microbiomes in stem (modularity index 0.438) and leaf (modularity index 0.448) microhabitats demonstrated more pronounced modular structures than those in roots (modularity index 0.427) and rhizosphere (modularity index 0.421) microhabitats (Figure 4B). Notably, the seeds-P exhibited the highest values for network modularity, average clustering coefficient, and average path length, indicative of structured, strongly correlated, and intricate microbiome networks (Figure 4). Conversely, daughter seeds displayed relatively lower values for these metrics, suggesting more fragmented and less-correlated microbiome networks. Among all assessed microhabitats, the ratio of positive to negative connections (i.e., edges) was lowest in leaf microhabitats (1.47) and highest in rhizosphere microhabitats (5.15), highlighting the varying degrees of ecologically significant effects on bacterial interactions across different microhabitats (Figure 4B).

Microbiomes often involve microorganisms that serve as central taxa, commonly referred to as central nodes, within their networks. These central nodes include modular centers and links, and their identification involves assessing the intra-module degree (Zi) and inter-module connectivity (Pi) of each ASV within the network. Across all networks, 279 connections, 35 module centers, and two network hubs were observed (Supplementary Figure 4 and Supplementary Table 5). The stem and leaf networks indicated a comparatively higher number of central nodes, with 138 and 110 nodes, respectively. In contrast, offspring seed and seed-P networks exhibited a notable difference in the central node count, featuring only 1 and 30 nodes, respectively (Figure 4B and Supplementary Table 5). Rhizosphere, stem, and leaf networks predominantly featured *Bacilli* as the major taxa. Furthermore, the stem and leaf networks identified crucial taxa (Supplementary Table 6), demonstrating the diverse ecological significance of different bacterial class within various cotton regions. Despite the limited number of central nodes in the seed network, these nodes exhibited the highest mesocosm centralities. This observation highlights their pivotal role in maintaining network structure stability, as indicated in Supplementary Table 5.

### Endophytic microbiomes of cotton seed cores

Cotton microbiomes exhibit distinct ecological niche-dependent dynamics. However, a consistent set of core microbiomes within cotton seeds remains prevalent, demonstrating their relative stability and resilience to external environmental influences. To comprehensively explore the composition and characteristics of this endophytic core microbiome, we synthesized and analyzed extensive data from diverse cotton genotypes and microhabitats.

**FIGURE 5:**
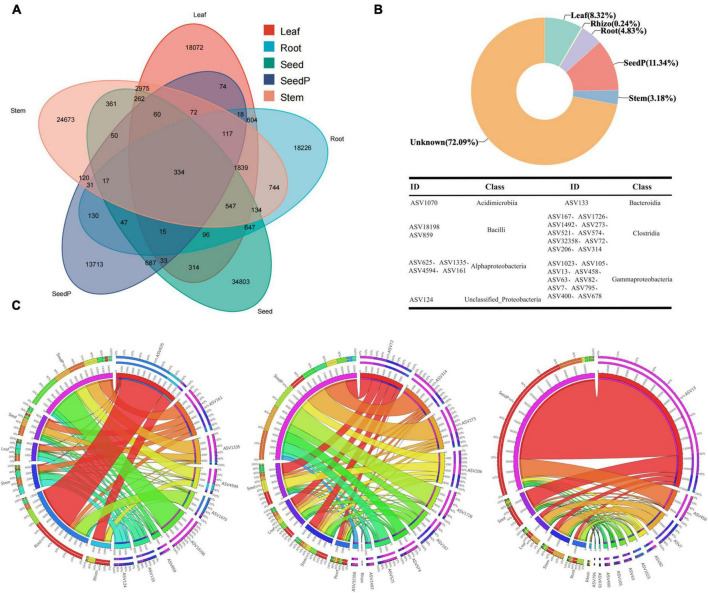
Core cotton endophytic microbiota and vertically transmitted taxa. **(A)** The Venn diagram shows that 334 ASVs coexist in the cotton endosphere compartments (root, stem, leaf, and parental progeny seed) at a frequency threshold of 70%. **(B)** SourceTracker analysis was used to analyze the contribution and trace the origin of endophytic bacteria in the offspring seeds. Combined with the Venn diagram, it was found that 26 ASVs are vertically transmitted from the parental seeds (seed-P) to the offspring cotton endosphere (endo) at a frequency threshold of 100%. The taxonomy of the ASVs is listed in Supplementary Table 7. **(C)** Circos plot shows the distribution proportion of core vertically transmitted microbiota among different plant compartments. Rhizo, Root, Stem, Leaf, Seed, and SeedP represent rhizosphere soil, root endosphere, stem endosphere, leaf endosphere, progeny seeds, and parent seeds, respectively. Detailed data is presented in Supplementary Table 7.

The study results revealed the presence of 23,546, 32,336, 25,432, 15,518, and 38,407 ASVs with frequencies exceeding 70% in samples obtained from the roots, stems, leaves, seeds, and seeds-P, respectively (Figure 5A). Particularly noteworthy was the identification of 1,839 shared ASVs in the Venn diagram among the root, stem, and leaf tissues, constituting 7.81%, 5.69%, and 7.23% of the ASVs within each tissue. In addition, 130 ASVs overlapped with the progeny seed roots, accounting for 0.84% of the total progeny seed ASVs. Meanwhile, 687 ASVs overlapped with the progeny seeds, representing 1.79% of the total progeny seed ASVs (Figure 5A and Supplementary Table 7). SourceTracker software and Bayesian algorithm-based analysis further elucidated the potential sources of microbiomes in the progeny seeds. Based on the microbiome structure of the source and sink samples, predictive analysis indicated that the largest proportion (11.34%) of ASVs in progeny seeds originated from seeds-P, whereas roots, stems, and leaves contributed 4.83%, 3.18%, and 8.32%, respectively. ASVs from the rhizosphere environment accounted for only 0.24% of the total ASVs, while the source of the remaining ASVs was unknown, comprising 72.09% (Figure 5B). Collectively, 334 core ASVs were successfully identified in five major cotton endophytic tissues (roots, stems, leaves, seeds and seeds-P), constituenting the core cotton endophytic microbiome. These core ASVs represented 0.87% of the relative abundance of progeny seeds. Further traceability analysis identified 29 core ASVs unique to progeny seeds distributed across seven microbiome classes, including *Acidimicrobiia*, *Alphaproteobacteria*, *Bacilli*, *Bacteroidia*, *Clostridia*, *Gammaproteobacteria*, and unclassified_*Proteobacteria* (Figures 5A, B and Supplementary Table 7). Among the core endophytic microbiome, *Clostridia* and *Gammaproteobacteria* were the most enriched taxa, each featuring 10 associated sequence variants (ASVs, Figure 5B). Remarkably, these common ASVs constituted 8.68% of the core cotton-endophytic microbiome. In particular, ASV_1070 of the *Acidimicrobiia* class exhibited substantial absolute abundance and a high occurrence frequency in the root microhabitat, surpassing other microhabitats (Figure 5C). Furthermore, three genera, *Bacilli*, *Clostridia*, and *Gammaproteobacteria*, exhibited significantly higher absolute abundance and frequency of occurrence in seeds-P than in other microhabitats, followed closely by seeds, while they were relatively scarce in rhizosphere microhabitats. These observations reinforced the hypothesis that the 29 ASVs, spanning seven different genera of microorganisms, were potentially transmitted through vertical pathways. This finding indicates selective transmission of only a few specific microorganisms during vertical transmission, suggesting a selective element in the transmission mechanism (Cordovez et al., 2019).

### Predictive functional characterization of cotton bacterial communities

**FIGURE 6:**
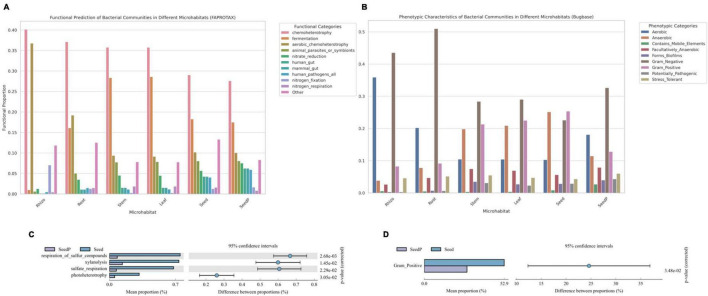
Functional predictions of microbial communities across various cotton microhabitats. **(A,C)** Bar charts depicting the FaproTax functional composition in different cotton microhabitats and the differences in microbial functions between parent and offspring seeds (Top 10 in functional abundance; *P*-value: 0.05). **(B,D)** Bar charts illustrating the phenotypic functions in different cotton microhabitats and the differences in microbial functions between parent and offspring seeds as predicted by BugBase (Top 10 in functional abundance; *P*-value: 0.05).

Comprehensive analyses using functional prediction tools such as FAPROTAX and Bugbase, which reference the SILVA rRNA and Greengenes databases, were employed to preliminarily explore the metabolic potential and phenotypic characteristics of microbiomes within various microhabitats of cotton (*Gossypium* spp.). These tools, based on 16S rRNA gene sequence analysis, aim to provide an initial understanding of the overall functional trends of microbiomes. Our findings indicate that the microbiome associated with roots exhibits heightened nitrate reduction capacity (Figure 6A), a crucial metabolic function for enhancing plant growth (Guilherme et al., 2019). Similarly, microbiomes in seeds-P demonstrated increased activities related to sulfur oxidation reduction and iron reduction (Figure 6C), potentially influencing their growth and survival in soil (Nakajima et al., 2019; Malviya et al., 2022). Moreover, microbiomes in microenvironments other than the rhizosphere showed higher activities in fermentation functions and anaerobicity (Figure 6B). A notable change was observed in the relative abundance of Gram-positive bacteria between seeds-P and seeds (Figure 6D), potentially reflecting an adaptation process that confers a survival advantage to Gram-positive microorganisms in seeds. Significant variations were also noted in various microbiome activities between seeds-P and seeds, including sulfate respiration, sulfide respiration, xylan degradation, and photoheterotrophy, potentially signifying shifts in intergenerational adaptation, impacting the viability and functional behavior of microorganisms with diverse roles in seeds (Moreira and Filho, 2016; Couturier et al., 2018; Graham et al., 2018).

The integrated analysis of these functional predictions provides preliminary insights into the functional mechanisms and ecological characteristics of microbiomes in various ecological niches of cotton plants, but should be viewed with caution once it is based only on predictions from short region of 16S rRNA gene sequences and may vary greatly among taxonomic groups and intra-specifically. These findings reveal the potential contributions of microbiomes to plant growth and health, while highlighting significant biological alterations that may occur across generations. Such changes could affect the overall stability and functionality of microbiomes, thereby impacting the ecological role of cotton in its environment. Further research is warranted to explore the accuracy of these functional predictions and to gain a deeper understanding of the potential long-term ecological implications for both plants and ecosystems.

## Discussion

This study aimed to comprehensively analyze the diversity, structure, and functional properties of microbiomes inhabiting different microhabitats within cotton (*Gossypium* spp.). These microorganisms engage in diverse beneficial interactions with host plants, including mutualistic symbiosis, co-evolution, and benign parasitism (Hardoim et al., 2008; Kaga et al., 2009). In addition, host plants can transfer beneficial endophytic microorganisms vertically to their progenies (Mastretta et al., 2009; Compant et al., 2010). To identify crucial vertically transferred taxa within the cotton microbiome, we analyzed microorganism populations originating from five distinct microhabitats across the three cotton genotypes. High-throughput 16S rRNA gene sequencing and diversity assessments were employed to elucidate the collective influence of different microhabitats (e.g., rhizosphere, root, stem, leaf, seed and seed-P) and cotton genotypes on microbiome diversity and structure. Our findings revealed that microhabitats exerted a more substantial influence on microbiome diversity than genotypes. This is consistent with prior research, such as the study of rice microbiome, which identified microhabitats as the primary determinant rather than environmental factors or host genotypes (Zhang et al., 2022). Furthermore, this study demonstrated distinct patterns of microorganism population dynamics in different cotton tissues. Analysis of microbiome diversity and network interactions revealed a significant decrease in both microbiome diversity and network complexity from roots, stems, and leaves to seeds. This trend may suggest a selective effect by the plant host, preferring to vertically transmit beneficial microorganisms to its progeny (Jeong et al., 2021; Zhang et al., 2022). Utilizing a variety of bioinformatics approaches, we conducted a detailed analysis of the microbiomes associated with cotton plants, aiming to define their core microbiome and to thoroughly investigate the vertical transmission of endophytic bacteria in seeds. In this study, we particularly noted the significant impact of environmental changes on the microbiome structure between seeds-P and seeds. By comparing the microbiome composition of seed-P and seeds, we revealed the dynamics of vertical transmission of microbiomes within the cotton lifecycle. This finding suggests that the adaptability and evolution of microbiomes are not only related to the genetic traits of the plant itself but may also be significantly influenced by external environmental factors. Subsequently, we generated predictions regarding the functional characteristics of microbiomes in each microhabitat, thus contributing to our understanding of the potential interactions between plants and microorganism populations. The results of this study offer a comprehensive and validated body of knowledge, enhancing our understanding of the vertical transfer of microbiomes within seeds and shedding light on the co-evolutionary dynamics between hosts and microorganisms.

*Alphaproteobacteria* and *Bacilli* indicated noteworthy dynamics across diverse microenvironments within cotton. This study revealed that *Alphaproteobacteria* exhibited a widespread distribution in the rhizosphere environment with a high abundance, indicating their specific adaptation to external conditions. These microorganisms are involved in a wide array of biological activities such as nitrogen fixation, release of biotrophic factors, pathogen antagonism, and mineral bioactivation (Dedysh et al., 2004; Taulé et al., 2012; Castanheira et al., 2014; Wicaksono et al., 2023). Bacterial communities were notably enriched within seeds across various cotton microhabitats, which is consistent with previous studies of cotton seed microbiomes (Adams and Kloepper, 2002). The ability of *Bacillus* microorganisms to produce stress-tolerant spores adapted to extreme environments may elucidate their successful colonization within seeds, potentially contributing to seed resilience to adverse conditions and pathogenic threats. Additionally, this trait may induce vertical transmission between plants and microorganisms (Gagné-Bourque et al., 2015), suggesting that *Bacillus* could employ seeds as a medium for persistent ecological impacts throughout the plant lifecycle. At the class level, *Clostridia*, *Bacteroidia*, and *Bacilli* were identified as the abundant resident microorganisms in cotton. *Clostridia* and *Bacteroidia* are more crucial for cotton ecosystems than *Bacilli*. *Clostridia* are pivotal in anaerobic decomposition and maintenance of soil structure, potentially promoting the health and stability of plant root systems (Mowlick et al., 2013; Huang et al., 2019). *Bacteroidia* primarily contribute to nutrient uptake and plant health and may play a role in plant disease resistance and nitrogen fixation processes (Pan et al., 2023). These microbiome taxa potentially exert significant functional impacts on the ecological stability, growth, and adaptation to adversity in cotton seeds. Therefore, a comprehensive exploration of the distribution and functional attributes of these microorganisms across different growth stages and cotton tissues is essential for gaining deeper insights into their integrated roles within the cotton ecosystem. Within the *Clostridia* class, the relative abundance of *Lachnospiraceae* increased significantly from the rhizosphere to the seed microenvironment. Notably, *Bacillus* spp. accounted for 10.36% of the seed microbiome. Previous studies tracking bacterial titers in seedlings and employing scanning electron microscopy have demonstrated the presence of *Bacillus* at high titers in stems, leaves, and pods of oilseed rape plants after 1 month of initial treatment (Reva et al., 2004). The ability of *Bacillus* to propagate vertically between plants through seeds was further confirmed by the isolation of several strains originating from seedlings. Overall, our results substantiate the persistence of cotton seed endophytes within seeds and validate the theory of their uniform distribution throughout plant growth, including intra-tissue migration.

The core endophytic microbiome of seeds can engage in co-evolutionary processes with their host plants over the course of the plant life cycle and develop efficient dispersal mechanisms across multiple generations (Truyens et al., 2014; Frank et al., 2017; Shade et al., 2017; Berg and Raaijmakers, 2018; Nelson, 2018). Therefore, identifying core microbiome groups is of critical importance for comprehending stable and consistent constituents within complex microbiomes (Shade and Handelsman, 2012). Core taxa, discerned through network analysis, such as *Bacilli* and *Cytophagales*, can be pivotal ‘bridges’ connecting microbiomes with host plants. The presence and activity of these core taxa can significantly affect the overall stability and functionality of the microbiome networks (Berg, 2009; Schmidt et al., 2019; Ikeda et al., 2023). In this study, through in-depth analysis of microbiomes, we identified seven core bacterial classes residing within cotton seeds: *Acidimicrobiia*, *Alphaproteobacteria*, *Bacilli*, *Bacteroidia*, *Clostridia*, *Gammaproteobacteria*, *and unclassified_Proteobacteria*. The core bacterial classes identified in this study may possess critical biological functions related to soil nutrient cycling, plant growth promotion, and disease resistance. For instance, within the *Alphaproteobacteria* class, four genera: *Bradyrhizobium*, *Azospirillales*, *Brevundimonas*, and *Allorhizobium_Neorhizobium_Pararhizobium_Rhizobium* have been noted. The genus *Bradyrhizobium*, known for its significant role in nitrogen fixation, might play a vital part in enhancing soil nitrogen content surrounding cotton plants (Pitumpe Arachchige et al., 2020; Favero et al., 2022). The order *Azospirillales* is recognized for its plant growth-promoting capabilities and may play a crucial role in the development of cotton seeds (Zhao et al., 2022). Genera *Brevundimonas* and *Allorhizobium_Neorhizobium_Pararhizobium_Rhizobium* have demonstrated adaptability and ecological functions in extreme environments (Ding et al., 2021). In the *Bacteroidia* class, the genus *Bacteroides* is believed to play a significant role in the decomposition of organic matter, potentially facilitating the nutrient cycling in the soil surrounding cotton seeds, which is essential for cotton growth (Wang et al., 2016). Certain strains within the *Bacilli* class also exhibit significant nitrogen-fixing and phosphate-solubilizing activities (Gelfand and Ravcheev, 2016). These microbiome taxa not only contribute to the diversity of cotton seed ecosystems, but also possess the potential to promote cotton growth and enhance agricultural yields. *Gammaproteobacteria*, as identified in several studies, has been identified as a class of disease-resistant facultative bacteria with versatile functionalities, including microbiome growth inhibition, nitrogen fixation, phosphorus solubilization, iron carrier production, and phytohormone synthesis (Walterson and Stavrinides, 2015). Moreover, the genus *Bacillus* has been associated with the growth of various plant seeds, such as maize (Johnston-Monje and Raizada, 2011; Liu et al., 2012; Tkalec et al., 2022), rice (Mano et al., 2006; Zhang et al., 2019), and radish (Rezki et al., 2018), where it is the dominant genus in the endophytic microbiomes. Our observations revealed substantial enrichment of the genus *Bacillus*, which dominated the endophytic microbiomes of seeds during the transition from seeds-P to seeds. Comparable findings have been substantiated in other plant seeds, including *Arabidopsis thaliana* (Truyens et al., 2013), rice (Mano et al., 2006), and kidney beans (López-López et al., 2010). Cotton yellow wilt, often referred to as “cotton blight,” is a detrimental disease caused by the soil-borne pathogen *Verticillium dahliae* and has substantial adverse effects on cotton yield and quality (Wang et al., 2021). Notably, *Bacillus* strains belonging to the *Bacillaceae* family have demonstrated effectiveness in inhibiting the growth and propagation of *Ralstonia solanacearum*, providing a potential avenue for biocontrol to manage this challenging disease (Sussanti et al., 2023). Through functional prediction analysis, we discovered that bacterial communities within different microhabitats of cotton exhibit a variety of functional traits. For instance, bacterial communities in the rhizosphere microenvironment demonstrated significant nitrogen-fixing capabilities, whereas those closely associated with roots showed enhanced nitrate reduction capacity. Additionally, we observed noticeable differences in microbiome functions between seeds and their progenies, which may reflect the mutual selection between host and microorganisms, aiding the plant’s adaptation to diverse environmental conditions (Ferreira et al., 2008; Rudgers et al., 2009). These preliminary findings suggest a potential key role of the seed core microbiomes in establishing beneficial microbiome symbioses essential for the health of plant offspring (Truyens et al., 2014). To deepen our understanding of the interactions and co-evolutionary mechanisms between these endophytes and their host plants, future research should concentrate on the isolation and purification of these strains, coupled with comprehensive molecular-level investigations. This approach provides valuable insights into how these microorganisms establish and sustain beneficial symbiotic relationships within cotton seeds.

## Conclusion

This study provides an extensive perspective on microbiome dynamics within cotton seeds throughout their growth and developmental cycles by employing amplicon-based microbiome analysis. This revealed the efficient migration of seed-derived bacteria into both aboveground (e.g., stems and leaves) and belowground (e.g., roots) plant structures. The identification of core microorganisms intimately associated with the interior of cotton seeds, pivotal network nodes, and core microbiome that underwent vertical transmission provides a theoretical foundation for microbiome regulation aimed at enhancing plant adaptation.

Future research should involve the isolation and functional characterization of these core microorganisms, along with an exploration of host-related factors, including gene expression, phytohormones, and metabolites that could affect their distribution during seed germination, seedling growth, and various stages of plant maturity. These investigations can enhance our understanding of how host factors regulate the temporal dynamics of microbiomes and how they influence plant physiological processes. In summary, this study establishes a robust theoretical and practical basis for customized microbiome applications in agriculture, industry, and environmental sciences.

## Data availability statement

The datasets presented in this study can be found in online repositories. The names of the repository/repositories and accession number(s) can be found below: https://www.ncbi.nlm.nih.gov/, PRJNA1028990.

## Author contributions

CW: Data curation, Formal Analysis, Methodology, Writing – original draft, Software, Visualization. XZ: Data curation, Formal Analysis, Methodology, Writing – original draft. LD: Investigation, Methodology, Writing – original draft. YW: Data curation, Investigation, Writing – original draft. YR: Investigation, Writing – original draft. HY: Methodology, Writing – original draft. AW: Funding acquisition, Project administration, Writing – review and editing. YF: Investigation, Methodology, Writing – original draft. JY: Data curation, Investigation, Writing – original draft. RL: Project administration, Writing – review and editing.
