# Supplementary figures and images for: Vertical transfer and functional characterization of cotton seed core microbiome
Source: Front Microbiol. 2024 Jan 9;14:1323342. doi: 10.3389/fmicb.2023.1323342 (PMC10803423; doi:10.3389/fmicb.2023.1323342)

A

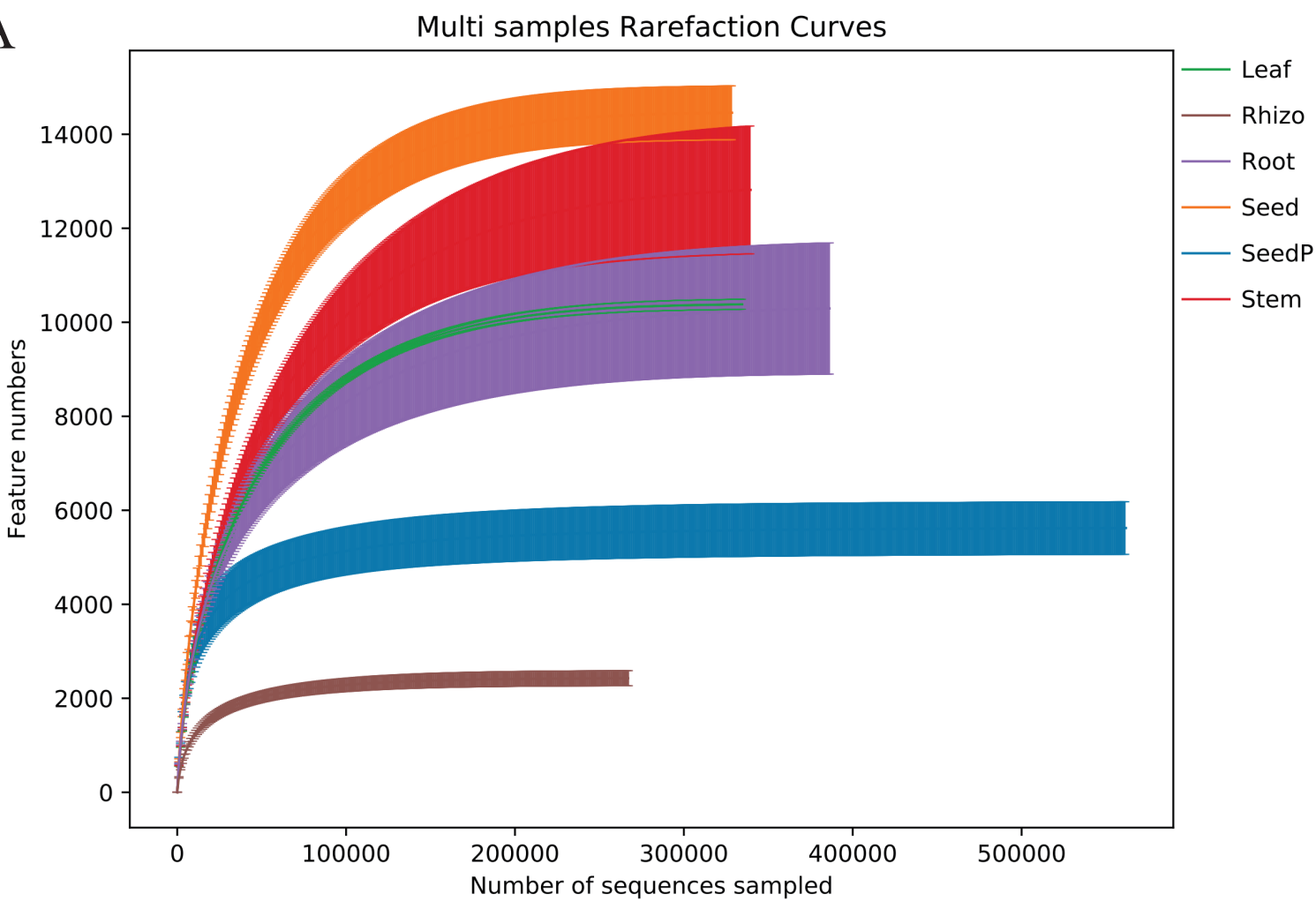

B

|        | AK (mg/kg)        | A-N(mg/kg)        | AP (µg/g )        | OM(g/kg)         | pH                | EC (µS/cm)        |
|--------|-------------------|-------------------|-------------------|------------------|-------------------|-------------------|
|        | F=4.5533;P=0.4478 | F=1.3178;P=0.3148 | F=0.0999;P=0.9059 | F=1.199;P=0.3454 | F=1.4099;P=0.2933 | F=0.5021;P=0.6212 |
| R20691 | 194.9183643       | 189.5893028       | 25.13829908       | 28.94139064      | 8.04              | 209               |
| R20692 | 199.3089562       | 182.2704837       | 27.96521765       | 27.58652773      | 8.21              | 120.8             |
| R20693 | 198.3603338       | 184.7230614       | 28.36903049       | 29.99508082      | 8.12              | 145.7             |
| R20694 | 196.8041237       | 189.9903007       | 29.8697004        | 27.56372324      | 8.11              | 165.8             |
| XLZ631 | 191.4327462       | 177.4269006       | 30.57103523       | 31.45666498      | 8.18              | 135.7             |
| XLZ632 | 199.1161129       | 182.8514457       | 30.08716174       | 30.68332584      | 8.08              | 168.9             |
| XLZ633 | 190.3013861       | 201.1650485       | 25.00562476       | 22.05979945      | 8.14              | 144.8             |
| XLZ634 | 195.6846759       | 218.529708        | 22.44506561       | 24.28625094      | 8.16              | 157.8             |
| XLZ781 | 192.6462545       | 175.6972112       | 29.3129393        | 22.41827768      | 8.19              | 158.6             |
| XLZ782 | 184.9825155       | 188.2033564       | 26.03481028       | 21.00589551      | 8.11              | 205.7             |
| XLZ783 | 218.9778122       | 170.8203125       | 27.70222244       | 28.79854369      | 8.22              | 178.5             |
| XLZ784 | 218.4430554       | 189.4594595       | 27.51370048       | 26.77248271      | 8.22              | 143.2             |

Supplement: Supplementary file 1 [file Image_1.pdf]

# Rhizo

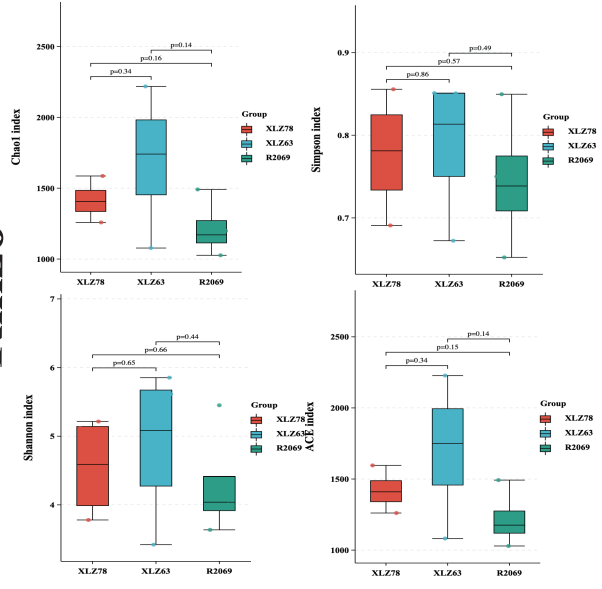

# Root

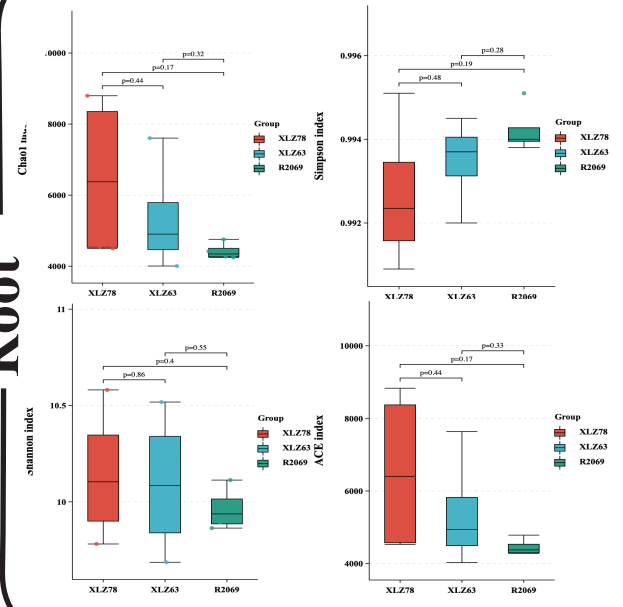

# Stem

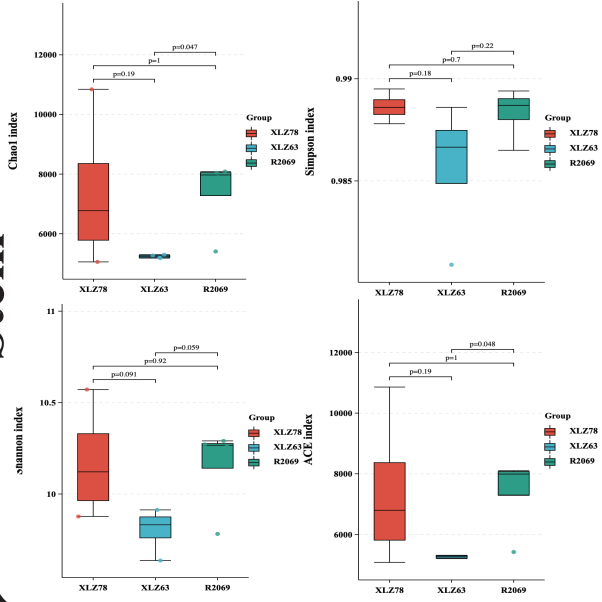

# Leaf

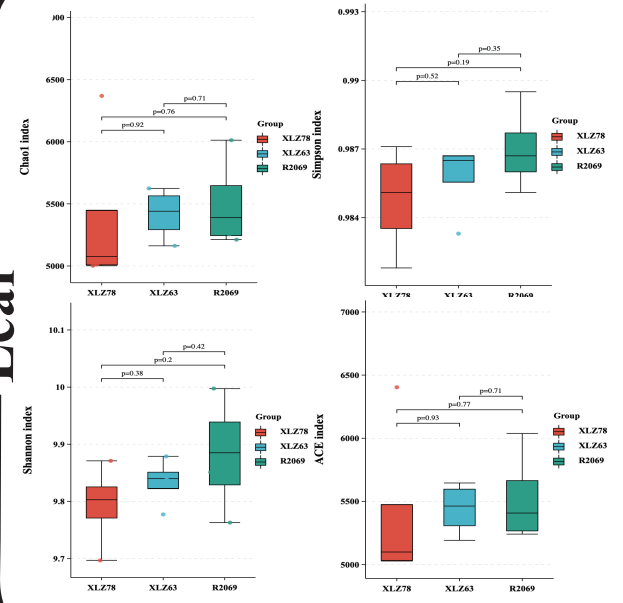

# Seed

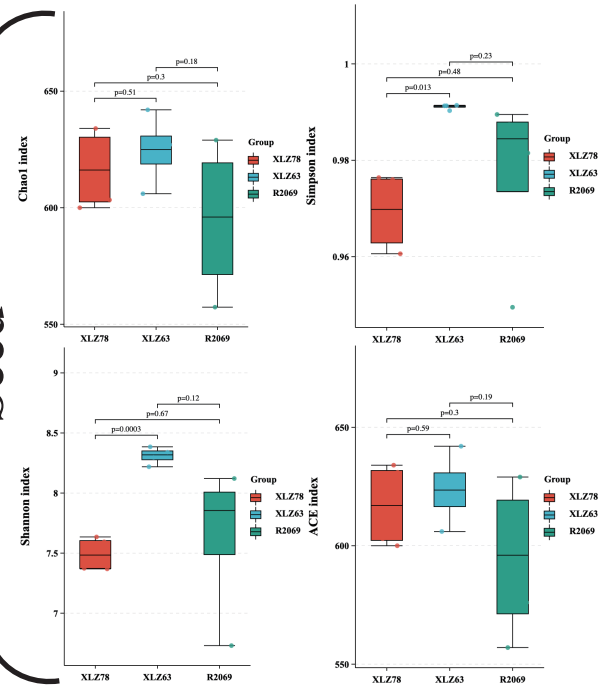

# Seed-P

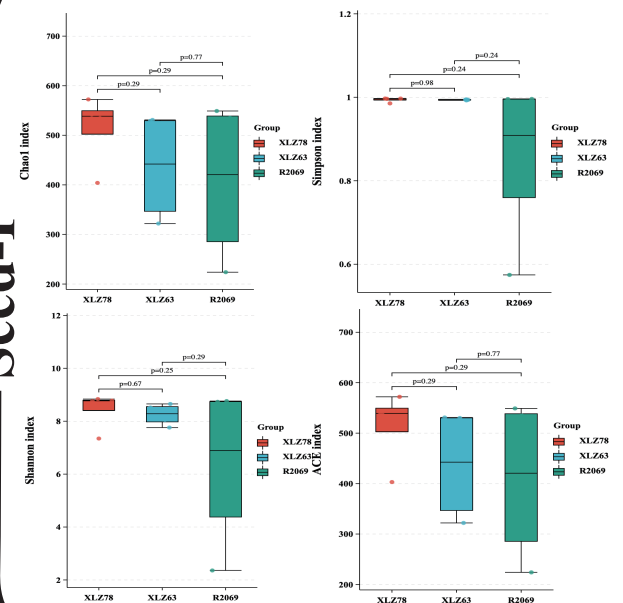

Supplement: Supplementary file 2 [file Image_2.pdf]

**A****Rhizosphere**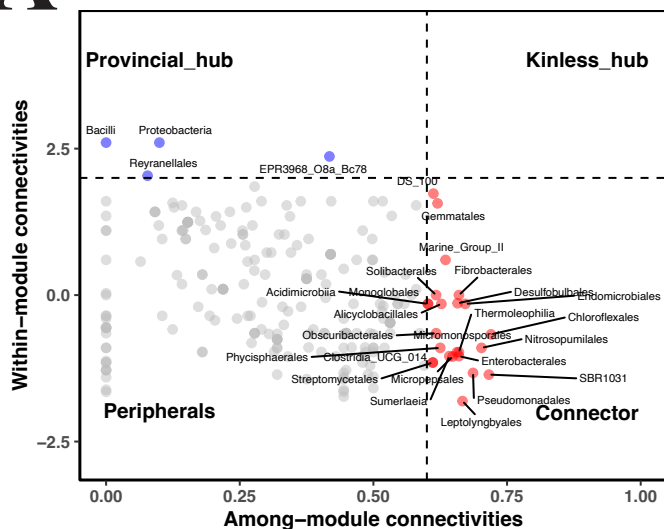**B****Root**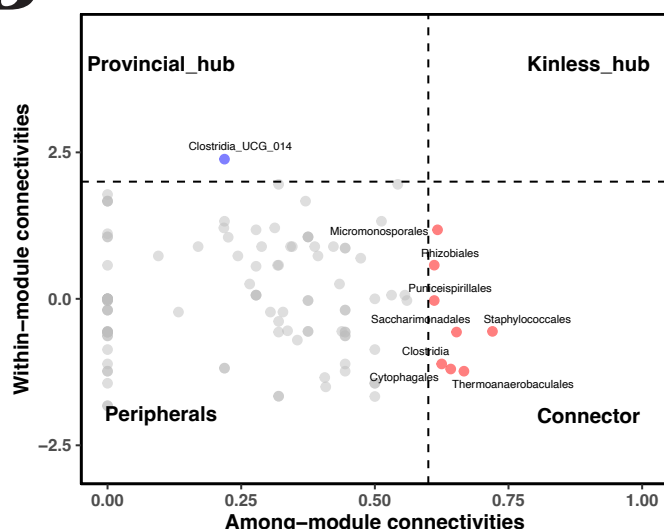**C****Stem**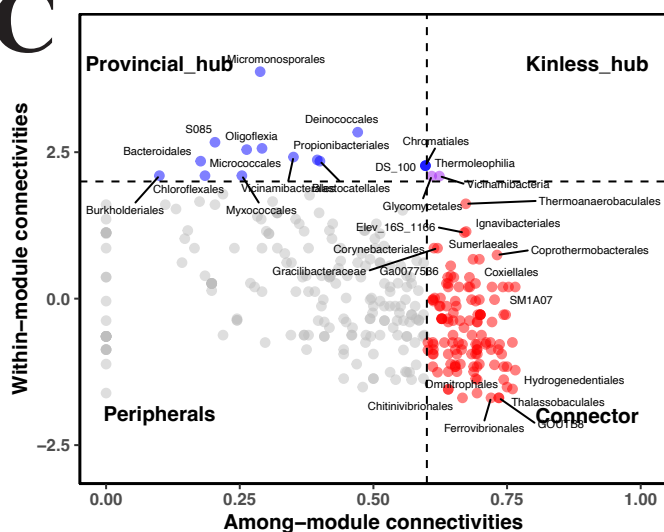**D****Leaf**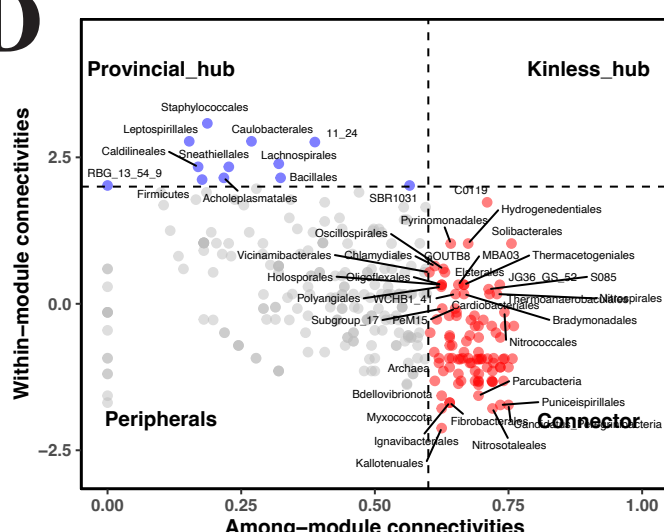**E****Seed**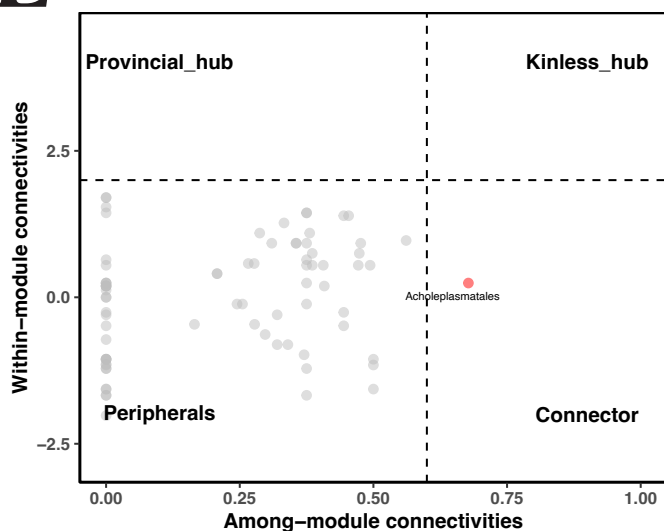**F****Seed-P**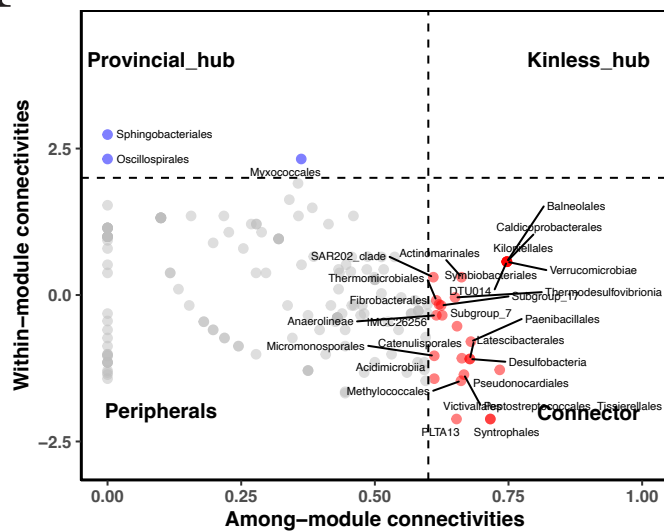

Supplement: Supplementary file 4 [file Image_4.pdf]
