# Supplementary material for: Vertical transfer and functional characterization of cotton seed core microbiome
Source: Front Microbiol. 2024 Jan 9;14:1323342. doi: 10.3389/fmicb.2023.1323342 (PMC10803423; doi:10.3389/fmicb.2023.1323342)
Supplement: Supplementary file 3 [file Image_3.pdf]

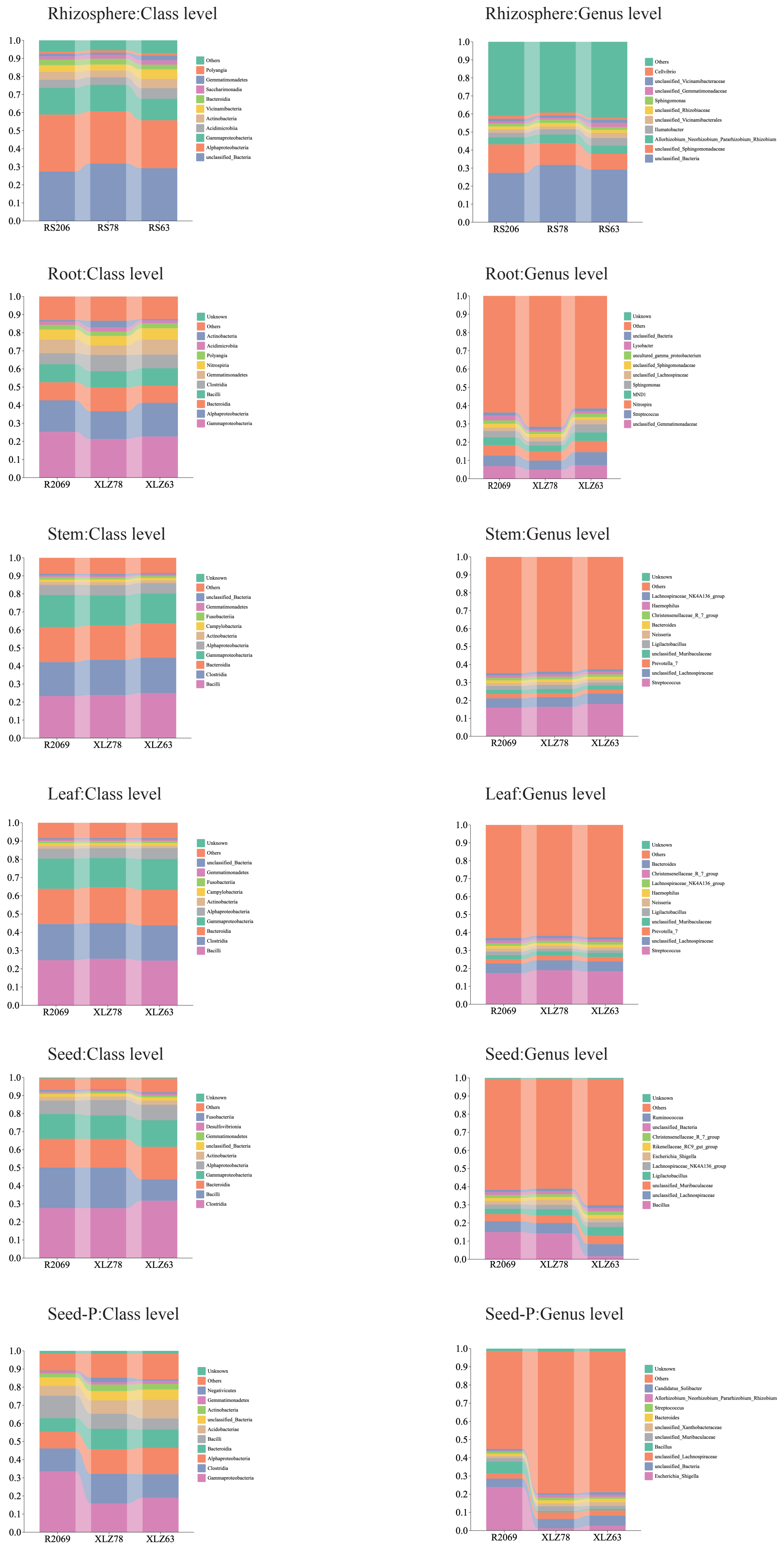
Rhizosphere:Class level
1.0
0.9
0.8
0.7
0.6
0.5
0.4
0.3
0.2
0.1
0.0
RS206
RS78
RS63
Others
Polyangia
Gemmatimonadetes
Saccharimonadia
Bacteroidia
Vicinamibacteria
Actinobacteria
Acidimicrobiia
Gammaproteobacteria
Alphaproteobacteria
unclassified_Bacteria
Rhizosphere:Genus level
RS206
RS78
RS63
Others
Cellvibrio
unclassified_Vicinamibacteraceae
unclassified_Gemmatimonadaceae
Sphingomonas
unclassified_Rhizobiaceae
unclassified_Vicinamibacterales
Ilumatobacter
Allorhizobium_Neorhizobium_Pararhizobium_Rhizobium
unclassified_Sphingomonadaceae
unclassified_Bacteria
Root:Class level
R2069
XLZ78
XLZ63
Unknown
Others
Actinobacteria
Acidimicrobiia
Polyangia
Nitrospiria
Gemmatimonadetes
Clostridia
Bacilli
Bacteroidia
Alphaproteobacteria
Gammaproteobacteria
Root:Genus level
R2069
XLZ78
XLZ63
Unknown
Others
unclassified_Bacteria
Lysobacter
uncultured_gamma_proteobacterium
unclassified_Sphingomonadaceae
unclassified_Lachnospiraceae
Sphingomonas
MND1
Nitrospira
Streptococcus
unclassified_Gemmatimonadaceae
Stem:Class level
R2069
XLZ78
XLZ63
Unknown
Others
unclassified_Bacteria
Gemmatimonadetes
Fusobacteriia
Campylobacteria
Actinobacteria
Alphaproteobacteria
Gammaproteobacteria
Bacteroidia
Clostridia
Bacilli
Stem:Genus level
R2069
XLZ78
XLZ63
Unknown
Others
Lachnospiraceae_NK4A136_group
Haemophilus
Christensenellaceae_R_7_group
Bacteroides
Neisseria
Ligilactobacillus
unclassified_Muribaculaceae
Prevotella_7
unclassified_Lachnospiraceae
Streptococcus
Leaf:Class level
R2069
XLZ78
XLZ63
Unknown
Others
unclassified_Bacteria
Gemmatimonadetes
Fusobacteriia
Campylobacteria
Actinobacteria
Alphaproteobacteria
Gammaproteobacteria
Bacteroidia
Clostridia
Bacilli
Leaf:Genus level
R2069
XLZ78
XLZ63
Unknown
Others
Bacteroides
Christensenellaceae_R_7_group
Lachnospiraceae_NK4A136_group
Haemophilus
Neisseria
Ligilactobacillus
unclassified_Muribaculaceae
Prevotella_7
unclassified_Lachnospiraceae
Streptococcus
Seed:Class level
R2069
XLZ78
XLZ63
Unknown
Others
Fusobacteriia
Desulfovibrionia
Gemmatimonadetes
unclassified_Bacteria
Actinobacteria
Alphaproteobacteria
Gammaproteobacteria
Bacteroidia
Bacilli
Clostridia
Seed:Genus level
R2069
XLZ78
XLZ63
Unknown
Others
Ruminococcus
unclassified_Bacteria
Christensenellaceae_R_7_group
Rikenellaceae_RC9_gut_group
Escherichia_Shigella
Lachnospiraceae_NK4A136_group
Ligilactobacillus
unclassified_Muribaculaceae
unclassified_Lachnospiraceae
Bacillus
Seed-P:Class level
R2069
XLZ78
XLZ63
Unknown
Others
Negativicutes
Gemmatimonadetes
Actinobacteria
unclassified_Bacteria
Acidobacteriae
Bacilli
Bacteroidia
Alphaproteobacteria
Clostridia
Gammaproteobacteria
Seed-P:Genus level
R2069
XLZ78
XLZ63
Unknown
Others
Candidatus_Solibacter
Allorhizobium_Neorhizobium_Pararhizobium_Rhizobium
Streptococcus
Bacteroides
unclassified_Xanthobacteraceae
unclassified_Muribaculaceae
Bacillus
unclassified_Lachnospiraceae
unclassified_Bacteria
Escherichia_Shigella
